# Lung Cancer Stem Cell Markers as Therapeutic Targets: An Update on Signaling Pathways and Therapies

**DOI:** 10.3389/fonc.2022.873994

**Published:** 2022-05-26

**Authors:** Yue Zheng, Laduona Wang, Limei Yin, Zhuoran Yao, Ruizhan Tong, Jianxin Xue, You Lu

**Affiliations:** ^1^ Department of Thoracic Oncology, Cancer Center and State Key Laboratory of Biotherapy, West China Hospital, Sichuan University, Chengdu, China; ^2^ Laboratory of Clinical Cell Therapy, West China Hospital, Sichuan University, Chengdu, China

**Keywords:** cancer stem cell, lung cancer, marker, signaling pathway, targeted therapy

## Abstract

Cancer stem cells, a relatively small group of self-renewing cancer cells, were first isolated from acute myeloid leukemia. These cells can play a crucial role in tumor metastasis, relapse, and therapy resistance. The cancer stem cell theory may be applied to lung cancer and explain the inefficiency of traditional treatments and eventual recurrence. However, because of the unclear accuracy and illusive biological function of cancer stem cells, some researchers remain cautious about this theory. Despite the ongoing controversy, cancer stem cells are still being investigated, and their biomarkers are being discovered for application in cancer diagnosis, targeted therapy, and prognosis prediction. Potential lung cancer stem cell markers mainly include surface biomarkers such as CD44, CD133, epithelial cell adhesion molecule, and ATP-binding cassette subfamily G member 2, along with intracellular biomarkers such as aldehyde dehydrogenase, sex-determining region Y-box 2, NANOG, and octamer-binding transcription factor 4. These markers have different structures and functions but are closely associated with the stem potential and uncontrollable proliferation of tumor cells. The aberrant activation of major signaling pathways, such as Notch, Hedgehog, and Wnt, may be associated with the expression and regulation of certain lung cancer stem cell markers, thus leading to lung cancer stem cell maintenance, chemotherapy resistance, and cancer promotion. Treatments targeting lung cancer stem cell markers, including antibody drugs, nanoparticle drugs, chimeric antigen receptor T-cell therapy, and other natural or synthetic specific inhibitors, may provide new hope for patients who are resistant to conventional lung cancer therapies. This review provides comprehensive and updated data on lung cancer stem cell markers with regard to their structures, functions, signaling pathways, and promising therapeutic target approaches, aiming to elucidate potential new therapies for lung cancer.

## 1 Introduction

Considerable achievements have been made in improving patient survival by providing better treatments. However, several obstacles remain because of drug resistance, metastasis, and the lack of specific targeted drugs. The cancer stem cell (CSC) theory suggested that there is a relatively small group of self-renewing cancer cells that play a crucial role in tumor metastasis, relapse, and therapy resistance (1). However, because of the unclear accuracy and illusive biological function of CSCs, some researchers remain cautious and find this theory controversial (2, 3). In lung cancer, CSC theory may also explain the inefficiency of traditional treatments and eventual recurrence. Despite the ongoing controversy, CSCs are still being investigated and discovered (4).

CSCs are a subtle group of tumor cells with potential multidirectional differentiation capacity, high self-renewal, and tumorigenicity (1). They were first isolated from acute myeloid leukemia (5, 6) and may be derived from either regular tissue-specific stem cells or differentiated cells at tumor initiation to activate survival pathways and gain the ability to proliferate indefinitely (7). Because of their importance, CSC biomarkers can be used in cancer diagnosis, targeted therapy, and prognosis prediction. Potential markers of lung CSCs mainly include surface biomarkers, such as CD44, CD133, epithelial cell adhesion molecule (EpCAM), and ATP-binding cassette subfamily G member 2 (ABCG2), along with intracellular biomarkers, such as aldehyde dehydrogenase (ALDH), sex-determining region Y-box 2 (SOX2), NANOG, and octamer-binding transcription factor 4 (OCT4). This review focuses on the most frequently studied lung CSC markers with their structures, functions, potential mechanisms, and signaling pathways of therapy resistance and cancer relapse. The objective of this review was to provide updated data on lung CSC markers as promising therapeutic targets in patients with lung cancer, hoping to bring new hope on lung cancer treatment.

## 2 Structure and Function of Lung Cancer Stem Cell Markers

Various possible lung CSC markers have been identified to label heterogeneous lung CSC populations. A summary of the potential biomarkers for lung CSC, including surface and intracellular markers, is discussed below. **Tables 1**, **2** provide a comprehensive list of the most frequent stem cell markers in non-small cell lung cancer (NSCLC) and small cell lung cancer (SCLC), respectively, and describe their structures and main functions. Notably, one marker is usually not enough to distinguish lung CSCs accurately. Thus, using marker combinations is a wise approach to identify and isolate cells that may stimulate cancer formation, chemoresistance, and recurrence.

**Table 1:** Potential non-small cell lung cancer stem cell markers, structures, and functions.

Stem cell markers	Structure	Function
Surface markers, CD		
CD44	Type I transmembrane glycoprotein (85–250 kDa)	Hyaluronic acid receptor
CD90	Glycosylphosphatidylinositol-anchored glycoprotein (25–35 kDa)	Cell-cell/environment communication
CD117	Type I transmembrane glycoprotein (approximately 145 kDa)	Tyrosine kinase growth factor receptor
CD133	Cholesterol-binding five-fold transmembrane glycoprotein (97–120 kDa)	Interacts with VEGF, participates in signal transduction
CD166	Type I transmembrane glycoprotein (100–105 kDa)	Activated leukocyte adhesion molecule
Surface markers, not CD		
EpCAM	Single transmembrane protein (30–40 kDa)	Cell adhesion, proliferation, differentiation, and migration
ABCG2	Half transporter (approximately 72 kDa) composed of six transmembrane domains and only one ATP-binding domain	Xenobiotic transporter and multidrug efflux pump related to chemoresistance
FZD	7-transmembrane protein (approximately 64 kDa)	Wnt signaling receptor
CXCR4	G protein-coupled seven-transmembrane protein (40–70 kDa)	Chemokine receptor
Intracellular markers		
ALDH	Polypeptide tetramer (50–55 kDa)	Alcohol metabolism; cell differentiation, drug resistance, and oxidative stress response
SOX2	Member of the SRY-related HMG box family (~2.4 kb)	Cell proliferation, apoptosis, EMT, tumor migration, invasion, and chemoresistance
OCT4	Member of the POU transcription factor family (16.4 kb)	Cell pluripotency, tumor metastasis, and therapy resistance
NANOG	DNA binding homeobox transcription factor (~150 kb)	Cell pluripotency, proliferation, and apoptosis
BMI1	A protooncogene	Cell proliferation, senescence, and tumor promotion

VEGF, vascular endothelial growth factor; EMT, epithelial-to-mesenchymal transition; EpCAM, epithelial cell adhesion molecule; ABCG2, ATP-binding cassette subfamily G member 2; FZD, frizzled receptors; PDZ, PSD-95, DLG, and ZO1; CXCR4, C-X-C motif chemokine receptor 4; ALDH, aldehyde dehydrogenase; SOX2, sex-determining region Y-box 2; SRY, sex-determining region Y; HMG, high-mobility group; OCT4, octamer-binding transcription factor 4; BMI1, B-cell-specific Moloney murine leukemia virus integration site 1.

**Table 2:** Potential small cell lung cancer stem cell markers, structures, and functions.

Stem cell markers	Structure	Function
Surface markers, CD		
CD24	Sialo-glycoprotein (30–70 kDa) anchored to the plasma membrane *via* a GPI link	Cell surface adhesion and signal transducing molecule
CD44	Type I transmembrane glycoprotein (85–250 kDa)	Hyaluronic acid receptor
CD87/uPAR	Highly glycosylated, GPI-anchored membrane protein (45–65 kDa)	uPA receptor; proteolysis regulation; cell adhesion, migration, and proliferation
CD90	GPI-anchored glycoprotein (25–35 kDa)	Cell-cell/environment communication
CD133	Cholesterol-binding five-fold transmembrane glycoprotein (97–120 kDa)	Interact with VEGF, participates in signal transduction
CD166	Type I transmembrane glycoprotein (100–105 kDa)	Activated leukocyte adhesion molecule
Surface markers, not CD		
EpCAM	Single transmembrane protein (30–40 kDa)	Cell adhesion, proliferation, differentiation, and migration
ABCG2	Half transporter (~72 kDa) composed of six transmembrane domains and only one ATP-binding domain	Xenobiotic transporter and multidrug efflux pump related to chemoresistance
PODXL1	Transmembrane glycosylated cell surface sialo-mucin (~55 kDa)	Sodium-hydrogen exchange regulatory cofactor 2; cell morphology and adhesion
PTCH	Patched gene (~70 kb) composed of 5 alternative first exons in addition to the other 22 exons	Cell differentiation and branching morphogenesis
Intracellular markers		
ALDH	Polypeptide tetramer (50–55 kDa)	Alcohol metabolism; cell differentiation, drug resistance, and oxidative stress response
SOX2	Member of the SRY-related HMG box family (~2.4 kb)	Cell proliferation, apoptosis, EMT, tumor migration, invasion, and chemoresistance
OCT4	Member of the POU transcription factor family (16.4 kb)	Cell pluripotency, tumor metastasis, and therapy resistance
BMI1	A protooncogene	Cell proliferation, senescence, and tumor promotion

GPI, glycosylphosphatidylinositol; uPAR, urokinase plasminogen activator receptor; uPA, urokinase plasminogen activator; VEGF, vascular endothelial growth factor; EMT, epithelial-to-mesenchymal transition; EpCAM, epithelial cell adhesion molecule; ABCG2, ATP-binding cassette subfamily G member 2; PODXL1, podocalyxin-like 1; PTCH, patched; ALDH, aldehyde dehydrogenase; SOX2, sex-determining region Y-box 2; SRY, sex-determining region Y; HMG, high-mobility group; OCT4, octamer-binding transcription factor 4; BMI1, B-cell-specific Moloney murine leukemia virus integration site 1.

### 2.1 Potential Surface Markers for Lung Cancer Stem Cell

#### 2.1.1 CD44

CD44 (P-glycoprotein 1), a type I transmembrane glycoprotein, belongs to the family of cell adhesion molecules, and its gene is located on human chromosome 11 (11p13). Human CD44 weighs 85–200 kDa and consists of 742 amino acids (8). Through selective shearing and post-translational modification, CD44 can form a variety of isoforms that can combine with a range of ligands on the cell surface and are involved in the physiological and pathological processes of cells. CD44 is a hyaluronic acid receptor, and the communication between CD44 and hyaluronic acid is speculated to induce cell shedding, metastasis, and invasion (9). CD44 is expressed in almost all types of tumors and is a potential marker of mesenchymal stem cells and CSCs (10). It was found that CD44^high^/CD24^low^ cells lost epithelial cell markers and expressed more mesenchymal cell markers. These cells achieved the ability of self-renewal and heterogeneous differentiation, demonstrating that epithelial-to-mesenchymal transition (EMT) may help normal and tumor cells acquire stemness (11). In lung cancer, studies have shown that CD44 expression is higher in NSCLC than in SCLC, and the highest expression level was observed in lung squamous cell carcinoma (12). Cells derived from NSCLC cell lines were found to have chromosomal aberrations and 1p36 deletion, which significantly reduce the expression of the tumor suppressor gene miR-34a located at 1p36. However, overexpression of miR-34a attenuates the stem phenotype of CD44+ cells (13). CD44 regulates several signaling pathways to promote cancer progression, including Notch, Hedgehog (HH), Wnt, STAT3, Hippo, JNK, and RhoGTPase, and is a coreceptor that mediates signaling pathways by receptor tyrosine kinases (14, 15). Besides, CD44 is a key mediator of adhesion between endothelial cells, thus playing a major role in pathological angiogenesis (16). By binding to ezrin, radixin and moesin proteins, CD44 can bind to the cytoskeleton with the cell membrane, which facilitates cancer cell growth and metastasis. CD44 can also mediate tumor proliferation and immune evasion by promoting PD-L1 expression on the tumor cell surface (17). CD44 expression may play a significant role in epidermal growth factor receptor (EGFR)-mutant neoplasms, particularly in early NSCLC (18, 19), and CD44 is important in predicting EMT upon EGFR-TKI monotherapy in patients with lung cancer (20). CD44 and ALDH co-expressing cells, which are higher in lung squamous cell carcinoma, always show increased self-renewal capacity, enhanced migration ability, and tumorigenicity (21).

#### 2.1.2 CD133

CD133 (prominin-1) is a cholesterol-binding five-fold transmembrane glycoprotein that was first isolated in 1997 from mouse neural and CD34+ human progenitor and hematopoietic stem cells (22). Subsequently, CD133 was identified as a potential CSC marker, and research on CD133 has been focused on lung, breast, colon, and liver cancers. The structure of CD133 contains one extracellular N-terminal, two intracellular loops rich in cysteine, two extracellular loops that possess nine N-linked glycosylation parts, and one cytoplasmic C-terminal. The human CD133 gene contains at least 37 exons, more than 150 kb in length, and is controlled by five promoters, encoding a 97–120 kDa glycoprotein composed of 865 amino acids (23).

In terms of its function in lung cancer, CD133 is not only a potential biomarker of lung CSC but also a potential therapeutic target and prognostic factor. CD133 correlates with metastasis, therapy resistance, and worse outcomes. CD133+ cells can expand, invade, and self-renew, whereas CD133- cells are terminally differentiated in lung cancer (24). Thus, targeting and destroying CD133+ lung CSCs may significantly inhibit tumor cell proliferation, migration, and invasion. However, the exact molecular mechanisms of CD133-related drug resistance remain elusive and require more attention. To date, evidence has shown that CD133 may regulate the growth and chemoresistance of CSCs by activating Wnt signaling, PI3K-AKT signaling, SRC-FAK signaling, interaction with EGFR, vascular endothelial growth factor autophagy, lipid metabolism, and low reactive oxygen species levels (25–27). In combination with other CSC biomarkers, such as CXCR4 (28) and BMI1 (29), CD133 is also positively correlated with EMT. And in hypoxic environment, up-regulation of OCT4 and SOX2 can induce CD133 expression in lung cancer cells (30).

#### 2.1.3 EpCAM

EpCAM, also known as CD326, is a type I transmembrane polypeptide and weighs approximately 40 kDa. EpCAM is composed of 314 amino acids with a long extracellular N-terminal, a single-spanning transmembrane domain, and a short intracellular C-terminal. The human EpCAM gene is approximately 14 kb and is located on chromosome 2 (2p21) (31). EpCAM is a potential biomarker for neoplasms of epithelial origin and a homotypic calcium-independent cell adhesion molecule. In lung cancer, EpCAM has been found to be a downstream target of metastasis-associated protein 1 (MTA1), and MTA1 overexpression can increase EpCAM levels and enhance tumor metastasis, leading to poor prognosis (32). EpCAM expression is also related to CD44 and CD166, and the triple-positive (EpCAM+/CD44+/CD166+) markers in NSCLC indicate higher self-renewal ability, clonal heterogeneity, and stemness-associated gene expression (33).

#### 2.1.4 ABCG2

ABCG2, also known as breast cancer resistance protein, is a 72 kDa xenobiotic half-transporter with 655 amino acids, and chromosome 4 (4q22) encodes the ABCG2 gene. ABCG2 consists of six transmembrane domains and only one ATP-binding domain, and it is detected in normal tissue cells and CSCs. Using cryo-electron microscopy, scholars have observed the high-resolution architecture of human ABCG2 and its interaction with inhibitory 5D3 antibody, suggesting its mechanism of multidrug recognition and transportation (34). ABCG2 is abundant in the side population phenotype in CSCs and effluxes Hoechst 33342 from cancer cells, associating ABCG2 with multidrug resistance (35). In lung CSCs, ABCG2 levels are elevated, and this may be related to the transcription factors Sp1 and Sp3, which can link with the promoter region of ABCG2 (36). Treatment of lung cancer cells with tobacco concentrates can lead to significant upregulation of ABCG2 and its transcription factors Sp1 and Nrf2, indicating that smoking may induce lung cancer partly through ABCG2 upregulation (37). Co-expression of ABCG2 and CD133 may lead to a higher risk of cisplatin resistance and tumor relapse (38).

### 2.2 Potential Intracellular Markers for Lung Cancer Stem Cell

#### 2.2.1 ALDH

ALDH is crucial in acetyl-coenzyme oxidation and differentiation regulation of normal stem cells. In NSCLC, ALDH levels are significantly enhanced and are involved in therapy resistance, mainly due to the increased expression of ALDH1A1 (39). ALDH1A1 is a 50–55 kDa protein sharing 501 amino acids, and the human ALDH1A1 gene is located on chromosome 9 (9q21.13). ALDH exists and functions as tetramer or dimer, and its monomers comprise the coenzyme binding, catalytic, and “arm-like” oligomerization parts (40). ALDH1A1 may be a potential CSC marker in solid tumors, and its expression is positively correlated with an epithelial-like phenotype in NSCLC (41). Compared with ALDH- cells, ALDH+ cells have greater self-renewal and tumorigenic capacity, and the level of ALDH1A1 correlates with poor survival. ALDH1A1 also correlates with Notch3, CD44, and CD133, which are related to chemoresistance and poor prognosis (21, 42).

#### 2.2.2 Transcription Factors

Transcription factors can drive cell migration and functional maturation, as well as control the multidirectional differentiation potency of embryonic stem cells and CSCs. They are the center of the cellular pluripotency regulatory network and control the transcription of pluripotency-related genes. In lung cancer, many studies have shown that intracellular transcription factors, such as SOX2, OCT4, and NANOG, are maladjusted and may therefore activate stemness genes and suppress differentiation genes.

SOX2 belongs to the sex-determining region Y-related high-mobility group box family and contributes to the maintenance of the pluripotency of embryonic stem cells and CSCs. SOX2 involves approximately 317 amino acids, and chromosome 3 (3q26.3) encodes the human SOX2 gene (43). SOX2 is a pleiotropic protooncogene related to stemness and EMT in lung cancer (44). It can regulate oncogenes, including c-MYC, Wnt1, Wnt2, and NOTCH1, and plays a major role in FGFR1-ERK1/2-SOX2 axis to stimulate metastasis (45). SOX2 can induce the expression of the tumor-related factors p63 and keratin 6 and suppress CDKN1A, which can rescue G1 cell cycle arrest in squamous cell carcinoma (46, 47), leading to cancer differentiation, migration, and invasion. In SCLC, SOX2 is crucial in the PIK3-AKT-SOX2 signaling pathway and may mediate chemoresistance (48). Genome-wide analysis has revealed that SOX2 amplification can drive the occurrence and development of SCLC.

OCT4, also known as POU5F1 or OCT3, is a member of the POU transcription factor family. Chromosome 6 (6p21.31) encodes the human OCT4 gene (POU5F1 gene) and has an average length of 16.4 kb. With multiple transcription initiation sites, it can transcribe different mRNA isoforms, thus translating into a variety of proteins (49). The translated protein contains an N-terminal domain that activates transcription, a conserved DNA-binding domain, a POU binding domain, and a C-terminal transactivation part. OCT4 plays a major role in maintaining and regaining pluripotency. In lung cancer, OCT4 expression is related to therapy resistance, cancer relapse, and worse outcomes (50). Studies have also shown that OCT4 promotes lung cancer progression by transcriptionally regulating the long non-coding RNAs NEAT1 and MALAT1 (51).

NANOG, a DNA-binding homeobox transcription factor, may promote cell proliferation, renewal, and stem properties (52). It has approximately 305 amino acids, and chromosome 12 (12p13.31) encodes the NANOG gene. NANOG protein is roughly divided into N-terminal, DNA-binding homeodomain, and C-terminal transcriptional activation part (53). In lung cancer, NANOG expression correlates with TNM stage, tumor differentiation, and survival (54). Thus, NANOG overexpression may be a promising target and predictive marker.

## 3 Major Signaling Pathways Related to Lung Cancer Stem Cell Markers

**Figure 1:**
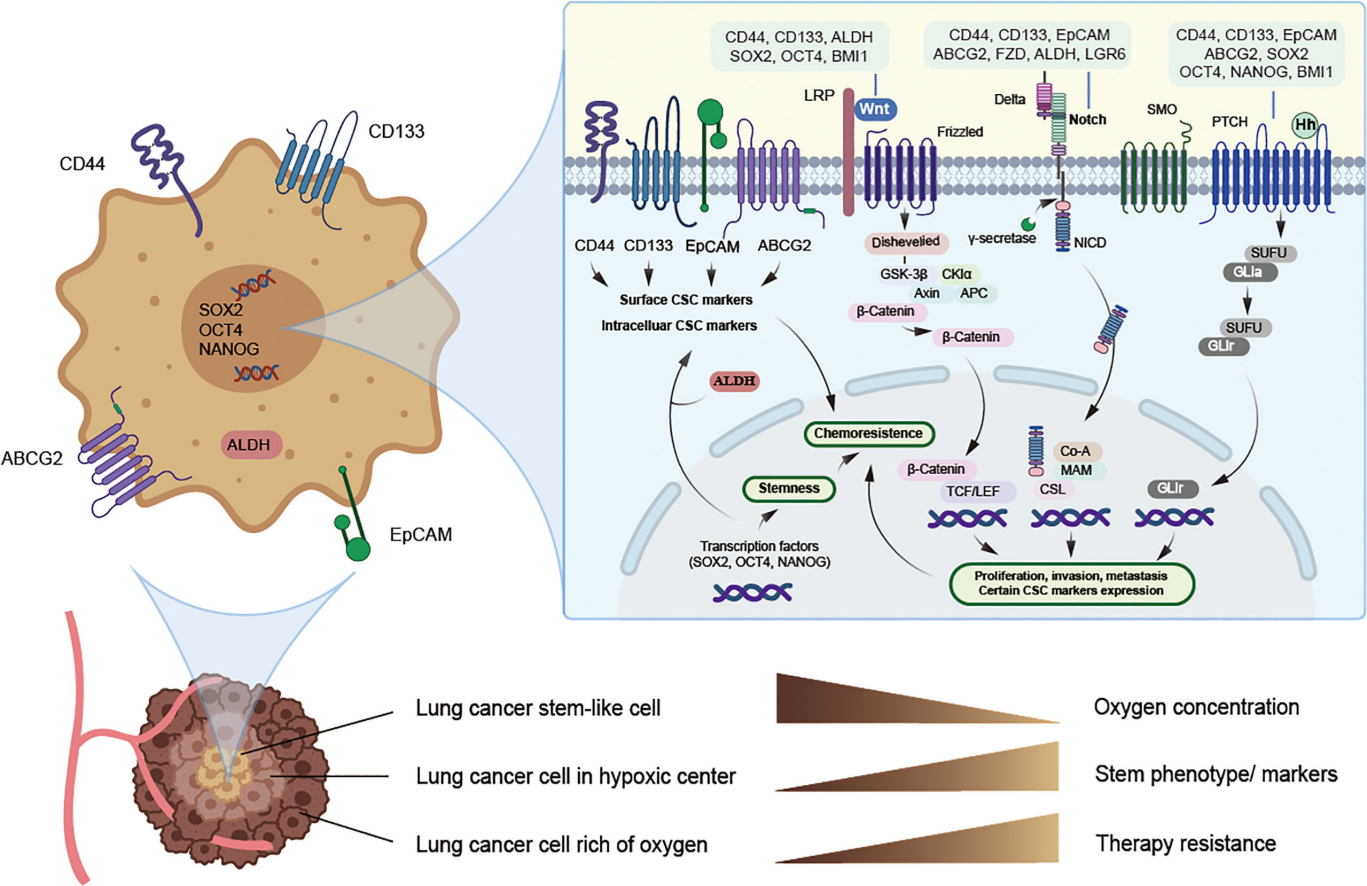
Potential lung cancer stem cell with its markers and major signaling pathways. CSCs can live deep in the hypoxic center of tumor and hypoxia may induce CSCs phenotype and the expression of CSC markers, thus promoting chemoresistance, while specific targeted therapy towards CSCs may solve this problem. Potential markers of lung cancer stem cells mainly include surface biomarkers, such as CD44, CD133, EpCAM and ABCG2, along with intracellular biomarkers, such as ALDH, SOX2, NANOG and OCT4, and they may act as promising therapeutic targets. Signaling pathways such as Notch, Hedgehog, and Wnt may jointly induce the expression of certain lung cancer stem cell markers and stimulate EMT, thus allowing tumor cells to obtain stemness and chemoresistance.

The ability of normal tissue stem cells to self-renew and differentiate is regulated by several signaling pathways, while the aberrant activation of these signals may be attributed to CSC maintenance, chemotherapy resistance, and cancer promotion. Certain biomarkers of lung CSCs are related and are partly regulated by these signals. The major signaling pathways currently found to be closely related to lung CSCs and their markers mainly include Notch, HH, and Wnt signaling (**Figure 1** and **Table 3**).

**Table 3:** Signals sustaining lung cancer stem cells and related stem cell markers.

Pathways	Function	Related stem cell markers
Notch signaling pathway	Control cell fate decisions and regulate self-renewal capacity	CD44, CD133, ALDH, SOX2, OCT4, BMI1
Hedgehog signaling pathway	Promote stem phenotype and cell proliferation	CD44, CD133, EpCAM, ABCG2, SOX2, OCT4, NANOG, BMI1
Wnt signaling pathway	Promote stem phenotype and cell proliferation	CD44, CD133, EpCAM, ABCG2, FZD, ALDH, LGR6
PI3K/AKT signaling pathway	Stimulate cell proliferation and inhibit apoptosis	CD44, CD87/uPAR, CD133, ABCG2, CXCR4, ALDH, SOX2, OCT4, NANOG
STAT3 signaling pathway	Regulate cell cycle and migration	CD24, CD44, CD133, ABCG2, CXCR4, ALDH, SOX2
Hippo signaling pathway	Regulate stem cell self-renewal, growth, apoptosis	CD44, CD133, EpCAM, ABCG2, SOX2, OCT4
JNK signaling pathway	Control cell proliferation, embryonic development, and apoptosis	CD44, CD133, ALDH, SOX2, NANOG

ALDH, aldehyde dehydrogenase; SOX2, sex-determining region Y-box 2; OCT4, octamer-binding transcription factor 4; BMI1, B-cell-specific Moloney murine leukemia virus integration site 1; EpCAM, epithelial cell adhesion molecule; ABCG2, ATP-binding cassette subfamily G member 2; FZD, frizzled receptors; LGR6, leucine-rich repeat-containing G-protein coupled receptor 5; uPAR, urokinase plasminogen activator receptor; CXCR4, C-X-C motif chemokine receptor 4.

### 3.1 Notch Signaling Pathway

Notch signaling is widely distributed in different cell types and plays a major role in cell communication, proliferation, differentiation, apoptosis, adhesion, and EMT. It is also conducive to mediating the crosstalk between the different compartments of the tumor microenvironment. Notch signals pass through Delta-like ligand 1 (DLL1), DLL3, DLL4, Jagged1, or Jagged2 on a cell to the Notch receptor paralog (Notch1, Notch2, Notch3, or Notch 4) on an adjacent cell, which releases the Notch intracellular domain and converts Notch receptors into downstream signal transducers (55). Notch signaling is critical for stem characteristics, and its abnormal activation may stimulate the growth and metastasis of tumor cells through its downstream proteins. The main regulatory genes include the HES family genes, the proto-oncogenes MYC, cyclin-dependent kinase inhibitor 1A, cyclin D3 human epidermal growth factor receptor 2, and Notch regulated ankyrin repeat protein (56). The HES family genes are transcriptional repressors that control stem cell characteristics. Genetic mutations in Notch can sustain the survival of CSCs and extend to all levels of NOTCH signal members (57).

Notch signaling is crucial in various neoplasms including leukemia, lymphoma, breast cancer, lung cancer, glioblastoma, and colon cancer. It is related to certain lung CSC markers, such as CD44, CD133, ALDH, OCT4, and SOX2 (58–60). In NSCLC, Notch 1 and Notch 2 are highly expressed (61, 62) and gain self-renewal by the transcription factor HES1. Notch 1 also protected lung CSCs from cisplatin-induced cell death through a pathway independent of HES1 (63). In addition to promoting the proliferation of NSCLC cells, the Notch signaling pathway can also mediate the metastasis of NSCLC through the circulatory and lymphatic systems. However, the function of abnormal Notch activation in SCLC is debated. Studies have shown that Notch signaling in SCLC is both a tumor suppressor and a pro-oncogene (64). Notch gene function loss and Notch activation inhibition in SCLC suggest that Notch signaling inhibits SCLC growth and that the Notch pathway can prevent the differentiation of precursor cells into neuroendocrine differentiation during lung development (65–67). However, in preclinical models, Notch signaling blockade combined with chemotherapy can inhibit tumor growth and delay tumor recurrence, which suggests a pro-carcinogenic part of Notch in SCLC (64, 68). In addition, Notch signaling blockade with shRNA against NOTCH or γ-secretase inhibitors can significantly suppress the growth of CD44+, CD133+, or ALDH+ tumor cells, consistent with a decrease in lung cancer cell growth and chemoresistance (58, 59, 69). Studies have also shown that cisplatin resistance induces the enrichment of CD133+ cells, which is partly due to the abnormal activation of Notch signaling, suggesting that this pathway is a potential target in regulating lung CSCs (59).

### 3.2 HH Signaling Pathway

The HH signaling pathway is crucial for embryonic development, and its abnormal activation is also associated with stem cell regulation and tumor occurrence. The HH ligands have various isoforms, including sonic HH, Indian HH, and desert HH, which can link with the transmembrane receptors Patched and Smoothened. Only when the protein family members maintain full length can they function as transcription promoters, activating the expression of downstream genes. After Patched and Smoothened transcription, released signal cascades contribute to the activation of glioma-associated oncogene (GLI) transcription factors, thereby driving HH target gene transcription and thus leading to better stem property maintenance (70).

Abnormal activity of HH signals is common in various solid tumors, such as medulloblastoma, glioma, myeloma, pancreatic cancer, esophageal cancer, gastric cancer, breast cancer, and lung cancer. Sonic HH and GLI-1 are highly active in the repair of lung airway epithelial injury. Both in SCLC and NSCLC, the expression of GLI-1 and sonic HH proteins was evaluated and associated with chromosome instability and stemness (71–73). The HH and Wnt pathways can jointly induce CSC biomarkers, such as CD44, CD133, BMI1, and LGR5, and promote EMT, thereby promoting tumor cell infiltration and distant metastasis and allowing tumor cells to obtain stem cell characteristics and drug resistance capacity (74). GLI-1 is also a strong regulator of SOX2 and develops resistance to EGFR inhibitors in NSCLC (75). Studies have found that phosphorylated SOX2 can be recruited to the hedgehog acyltransferase (HHAT) promoter to enhance HHAT levels, which is conducive for maintaining stemness. Activation of the PKC1-SOX2-HHAT signal axis is conducive to maintaining the stem function of primary lung squamous cells (76). Activated HH signaling is also significantly related to drug transporters, such as ABCG2, thus promoting chemotherapeutic drug resistance. This pathway may regulate several aspects of the lung CSC state and increase cancer metastasis. It is, therefore, not surprising that the inhibitors of this pathway offer great clinical hope.

### 3.3 Wnt Signaling Pathway

As a complex protein network, Wnt signaling is mostly found in the development of the central nervous system and mammalian embryos (77). However, it is also associated with abnormal processes in various tumors, such as breast cancer, colorectal cancer, liver cancer, leukemia, and lung cancer. The three primary Wnt pathways are the canonical Wnt pathway, which is involved in gene expression, the noncanonical Wnt/planar cell polarity pathway, which coordinates the cytoskeleton, and the noncanonical Wnt/calcium pathway, which adjusts the concentration of intracellular calcium (78). All three Wnt signaling pathways are activated by Wnt protein ligands binding to Frizzled receptors and coreceptors, such as low-density lipoprotein receptor-related protein 5 and 6, which transmit the signal to various intracellular proteins. Wnt ligands are secreted proteins, and this pathway is often paracrine or autocrine. The Wnt signaling pathway can regulate cell differentiation and proliferation, and is important in carcinogenesis, invasion, progression, and CSC maintenance (79). Hypoxia can activate Wnt signaling pathway, promote the non-differentiation and self-renewal ability of CSCs (80, 81). What’s more, tumor-associated fibroblasts in the environment can activate Wnt and Notch signaling pathways, produce metalloproteinases such as MMP2, MMP3 and MMP9 to reshape the extracellular matrix and promote self-renewal of EMT and CSCs (82). And some inflammatory microenvironments, which promote NF-κB and Wnt signaling, can induce tumor non-stem cells to acquire tumor-initiation capabilities (83).

In human lung cancer, the main Wnt ligands are Wnt1, Wnt2, Wnt5a, and Wnt7a, and an increase in Wnt proteins, Wnt1 or Wnt5a alone, is significantly related to poor prognosis in NSCLC (84). Studies suggest that β-catenin promotes the number of lung CSCs mainly by decreasing differentiation, rather than promoting direct stem cell proliferation (85). The mutation of β-catenin in tumor cells can cause itself to be inactivated by phosphorylation and degraded by ubiquitination. β-catenin accumulates in large amounts in the cytoplasm and enters the nucleus to regulate cell division and stem cell-related genes, leading to uncontrolled cell proliferation and tumorigenesis (86). Wnt signaling can regulate various lung CSC markers such as NANOG and OCT4, and the different Wnt microenvironments in different types of NSCLC lead to imparities in ABC transporter expression, such as ABCB1 and ABCG2 (87–89). Through the non-canonical Wnt pathway, Wnt5a can increase the stem properties of ALDH+ lung CSC in cisplatin-resistant NSCLC (90). In addition, Wnt signaling activation partly results from EMT, which is crucial in CSC reorganization and maintenance. EMT has been reported to induce β-catenin/E-cadherin/SOX15 conversion into β-catenin/Twist1/TCF4, thus inducing the transcription of the lung CSC marker ABCG2 (91). Cell subpopulations with nuclear high β-catenin, Twist1, CD133 together with low E-cadherin, and SOX15 can be used as diagnostic markers in lung cancer (91, 92).

## 4 Lung Cancer Stem Cell Markers as Therapeutic Targets

**Figure 2:**
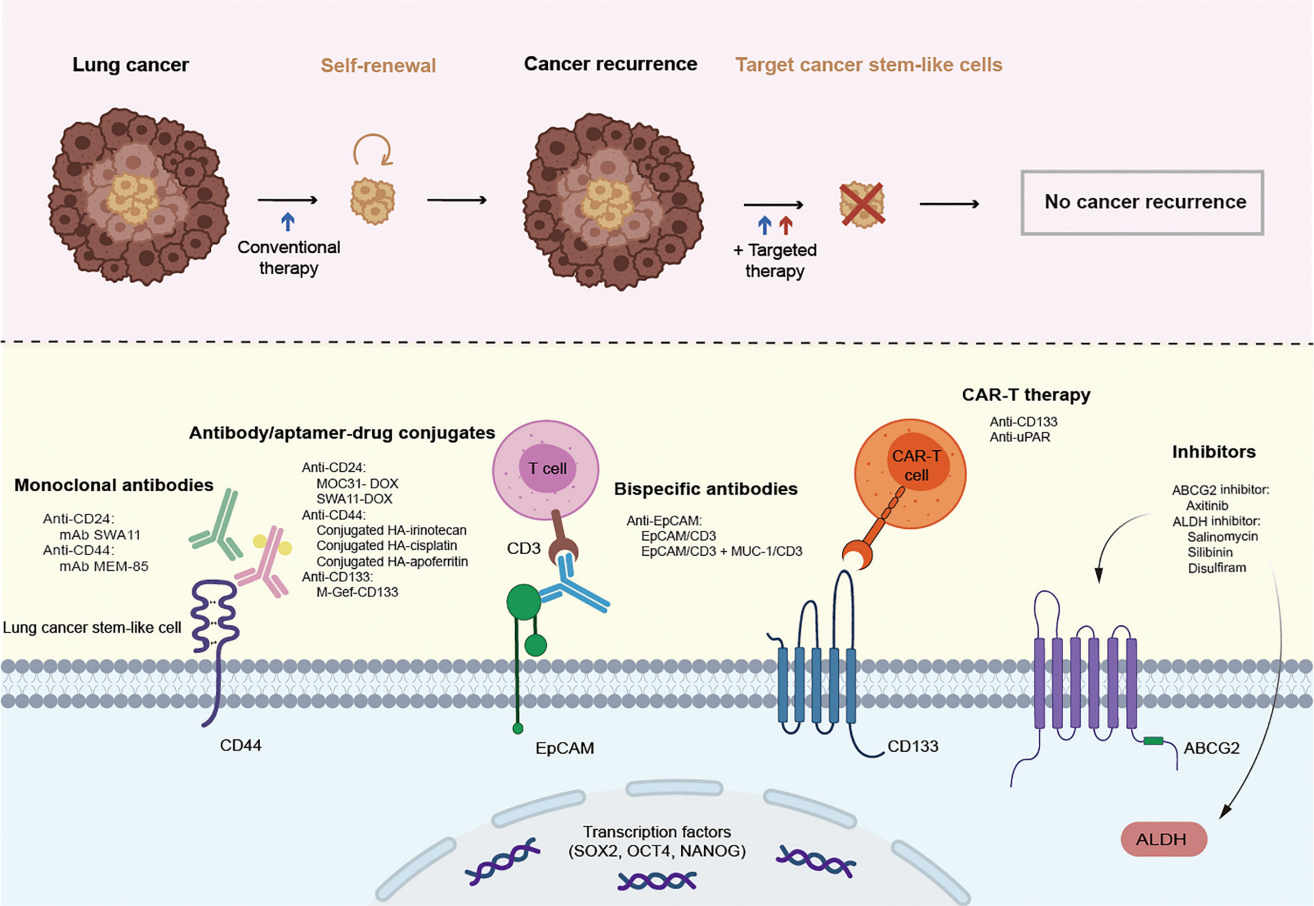
Promising therapies targeting lung cancer stem cell markers. CSCs are resistant to conventional therapy and lead to cancer recurrence, unless being specifically targeted. Therapies targeting lung cancer stem cell markers mainly rely on the specific recognition and binding of antibodies to lung cancer stem cell surface markers, thus leading to a variety of antibody drugs including monoclonal antibody, antibody-drug conjugate, bispecific antibody, and emerging nanoparticle drugs. In addition, novel CAR-T cell immunotherapy as well as inhibitors also play an increasingly important role in the experiments and future clinical trials.

Although great achievements have been made in improving survival in patients with lung cancer through conventional treatments and combinatorial therapies, concomitant congenital or acquired drug resistance still limits the therapeutic effect and leads to poor clinical outcomes. Targeting CSCs in the lung cancer microenvironment may be a promising therapy for patients who are currently resistant to traditional lung cancer treatments. From this perspective, lung CSC markers may not only help to identify and isolate these lung CSC subpopulations but may also be conducive to specific targeted therapies. Therapies targeting lung CSC markers mainly depend on the specific recognition and binding of antibodies to lung CSC surface markers, thus leading to a variety of antibody drugs and nanoparticle drugs. In addition, novel chimeric antigen receptor T (CAR-T) cell immunotherapy as well as inhibitors, such as natural compounds and siRNA knockdown, also play an increasingly important role in experiments and future clinical trials (**Figure 2** and **Table 4**). Besides, there are some novel clinical trials regarding lung CSCs targeting like CSC-loaded DC vaccines without posted outcome (ClinicalTrials.gov: NCT02084823^i^) and targeting lung CSCs expressing TRAIL which is still recruiting (ClinicalTrials.gov: NCT03298763^ii^). In the succeeding sections, we will provide update on and discuss promising data of lung CSC markers as therapeutic targets, as well as emphasize topics for future investigations.

**Table 4:** Potential therapies targeting lung cancer stem cell markers.

Approach	Target	Intervention
Monoclonal antibody	CD24	mAb SWA11
	CD44	mAb MEM-85
Antibody/aptamer-drug conjugate	CD24	MOC31- DOX, SWA11-DOX
	CD44	Conjugated HA-irinotecan
		Conjugated HA-cisplatin
		Conjugated HA-apoferritin
		Apt1-Lip
	CD133	Salinomycin sodium lipid-polymer hybrid nanoparticles
		Docetaxel liposome surface modified with CD133 aptamer
		M-Gef-CD133
	CD166	Probody drug conjugate CX-2009
	EpCAM	Drug-loaded nano- and EpCAM immuno-nanoparticles
	CXCR4	CXCR4 antagonist LFC131 conjugated PLGA nanoparticles
Bispecific antibody	EpCAM/CD3, MUC-1/CD3	EpCAM/CD3 BsAb with MUC-1/CD3 BsAb combined treatment
	EpCAM/CD3	MuS110
		Catumaxomab
Chimeric antigen receptor T cells	uPAR	Senescence-targeted CAR-T cells
	CD133	Combination of enhanced CD133-specific CAR-T, CD73 blockage and anti PD-1 therapy
Other inhibitors	ABCG2, CD117	Axitinib
	ALDH	Salinomycin
		Silibinin
		Disulfiram

mAb, monoclonal antibody; DOX, doxorubicin; HA, hyaluronan; Apt1, 2′-F-pyrimidine-containing RNA aptamer; Lip, liposome; M-Gef-CD133, gefitinib-loaded poly(ethylene glycol) 2000-distearoylphosphatidylethanolamine nanomicelles; EpCAM, epithelial cell adhesion molecule; CXCR4, C-X-C motif chemokine receptor 4; PLGA, poly lactic-co-glycolic acid; BsAb, bispecific antibody; CAR-T, chimeric antigen receptor T; uPAR, urokinase plasminogen activator receptor; ABCG2, ATP-binding cassette subfamily G member 2; ALDH, aldehyde dehydrogenase.

### 4.1 Antibody Drugs Targeting Lung Cancer Stem Cell Markers

In recent years, antibody drugs have become the main new drugs developed because of their high specificity and low adverse reactions. They have been approved for a variety of clinical applications, as well as in the field of CSCs. Targeted therapies occur when antibodies specifically recognize and bind to certain lung CSC surface markers or signaling pathways. Antibodies targeting lung CSC markers mainly include monoclonal antibody (mAb), antibody/aptamer-drug conjugate (ADC), bispecific antibody (BsAb), and emerging nanoparticle drugs.

Therapeutic mAbs are a class of antibody drugs that have high homogeneity and pharmacological effects against a single antigen epitope. mAbs are characterized by strong targeting, low toxicity, and relatively easy development, ranking the mainstream of biotechnology drug research for a long time. At present, many mAbs targeting lung CSC markers have been used in basic experiments or clinical trials; for instance, mAb SWA11 targeting CD24 (93) and MEM-85 targeting CD44 (94). In addition, mAbs targeting lung CSC markers may strongly alter the tumor microenvironment and retard tumor growth (95).

ADCs are a class of drugs that conjugate mAbs to different numbers of small molecular cytotoxins through chemical conjugates. Chemotherapy and radiotherapy, as important complementary approaches of surgical treatment, play an active role in cancer treatment. However, chemotherapy drugs and radioactive substances can not only act on tumor cells, but also accidently injure normal tissue cells, thus causing greater adverse reactions. Fortunately, ADC drugs solve this issue by wisely conjugating cytotoxic drugs or radioactive substances to mAb, in which way ADCs combine the advantages of strong targeting and high tumor cytotoxicity and reduce the side effects to innocent normal cells. ADC drugs have also become a research hotspot for lung CSC targeting treatment in recent years. Some ADC drugs targeting CD44, such as conjugation of hyaluronan with irinotecan (96), cisplatin (97), and apoferritin (98), are effective in reducing or even eliminating stem cells in lung cancer. Numerous studies have shown that ADCs targeting CD133 (99–101), CD166 (102), EpCAM (103), and CXCR4 (104) can effectively inhibit the growth of lung CSCs. Because of the existence of the blood-brain tumor barrier in mAb treatment, systemic drugs have very limited efficacy in treating brain metastases. To address this problem, targeted drugs loaded with nanomaterials have been designed to effectively solve this problem through cross-cell and cell bypass pathways (105, 106).

BsAbs are novel antibodies that bind two different antigens simultaneously and do not exist naturally. The modified Fab fragment in the BsAb can bind to the epitope of cytotoxic drugs or the surface antigen of immune cells. Thus, BsAbs can not only target cancer cells and prevent cytotoxic effects on normal tissues, but also link with NK cells or CD8 T cells, which may eradicate cancer cells through a direct killing effect. In NSCLC, researchers have found that c-MET/CTLA-4 BsAb-targeting lung CSCs have potential therapeutic effects (107). Catumaxomab (108–110) or MuS110 (111, 112) can target EpCAM and CD3 and potentially inhibit local cancer growth. Scientists then combined EpCAM/CD3 BsAb with MUC-1/CD3 BsAb and surprisingly found greater specificity and better outcome (113), showing a possible immunotherapeutic approach.

Targeted therapy with antibody drugs brings new light to conquer tumors and CSCs. However, similar to other therapies, several concerns exist regarding safety risks, such as off-target effects, allergies, and cytokine storm. Compared with other tumors, antibody drugs for lung CSCs are few, probably because of the scarcity of suitable targets. Under these circumstances, basic and clinical research should be conducted to find more specific and better antigens toward lung CSCs and develop new carrier materials to promote the entry of antibody drugs into cancer tissue.

### 4.2 CAR-T Therapies Targeting Lung Cancer Stem Cell Markers

CAR-T therapy is used to isolate T cells from patients. It uses genetic engineering technology to transduce the CAR gene that specifically recognizes a tumor cell antigen into T cells, expands *in vitro*, and finally transplants it into patients to exert tumor-killing effect. It is MHC-free and therefore avoids tumor escape due to reduced MHC expression in tumor cells. CAR is composed of three parts: the intracellular region, which is the signaling molecule that mediates T cell activation, transmembrane region, and extracellular region, which is the variable region of mAb that can bind specific tumor antigens. As an innovative and promising therapeutic strategy, CAR-T therapy has remarkable efficacy and safety in the treatment of hematological tumors (114). It has also shed new light on solid cancer treatment, including lung cancer. However, CAR-T therapy is confronted with difficulties in treating solid tumors, mainly because of the scarcity of real tumor-specific antigens and the heterogeneity of tumor-associated antigens on the surface of solid tumors. Therefore, selecting specific antigens as targets for CAR-T cells to treat solid tumors is important, and the promising experimental and clinical use of CAR-T cells still needs more attention and effort. Given the potential to identify surface stem cell markers or antigens, CAR-T has become an appealing practice to target CSCs specifically. uPAR-specific CAR-T cells have also been reported to prolong survival and relieve liver fibrosis in mice with lung cancer (115). In SCLC, CAR-T cells targeting CD133 can migrate to cancer lesions, kill cancer cells, and improve survival, but cannot eliminate cancer cells. Combining CD133-specific CAR-T, CD73 blockage, and anti PD-1 therapy can particularly eradicate lung CSCs and may contribute to curable influence in a terminal disease (116). From this perspective, we should exert more effort to create stronger CAR-T structures and find better specific targets and therapy combination strategies.

## 5 Conclusions

Lung CSC is a subtle group of lung cancer cells with potential multidirectional differentiation capacity, high self-renewal, and tumorigenicity. Potential markers of lung CSCs mainly include surface biomarkers, such as CD44, CD133, EpCAM, and ABCG2, along with intracellular biomarkers, such as ALDH, SOX2, NANOG, and OCT4. These markers have different structures, but they are all related to the stem potential and uncontrollable proliferation of tumor cells. The aberrant activation of major signaling pathways, such as Notch, HH, and Wnt signaling pathways, may be related to the expression and regulation of certain lung CSC markers. For patients who are resistant to conventional lung cancer therapies, treatments targeting lung CSC markers may bring new hope. These treatments mainly include antibody drugs, nanoparticle drugs, CAR-T therapy, and other natural or synthetic specific inhibitors.

Although lung CSC theoretically provides many new ideas, the challenges brought about by it cannot be underestimated. First, the known lung CSC markers may not only exist in lung CSCs, and they merely have reference significance. lung CSCs and normal stem cells have similar functions and pathways, which makes them difficult to distinguish from each other during treatment; thus, it is difficult to show a satisfying targeting effect. More studies are needed to explore more specific markers and more targeted therapies against lung CSCs. Second, the specific molecular mechanisms and detailed signaling pathways that each lung CSC is used to control and regulate stem cell phenotypes are not well understood. Third, there is a lack of suitable animal models of lung CSCs for study. Establishing a new animal model, such as real human lung cancer, is important to clarify the mechanism of malignant transformation from lung stem cells to lung CSCs. Fourth, the tumor environmental factors and interactions between lung CSCs and other cells are poorly understood. Finally, more innovative and powerful approaches for the therapeutic targeting of lung CSCs should be explored. CAR-T therapy in solid cancer is promising but does not go smoothly, and lung CSC surface marker-specific CAR-T is rare. Identifying better CAR-T-specific targets and combination therapy against lung CSCs is important. Only continuous in-depth exploration can update the treatment and improve the prognosis of lung cancer in the future.

## Author Contributions

The study idea was conceived by YL and YZ. YZ and LW were responsible for the collection and assembly of data. All authors were involved in the writing and final approval of the manuscript.

## Funding

This work was supported by 1·3·5 project for disciplines of excellence, West China Hospital, Sichuan University [ZYJC21003] and the National Natural Science Foundation of China [grants 82073336].

## Conflict of Interest

The authors declare that the research was conducted in the absence of any commercial or financial relationships that could be construed as a potential conflict of interest.

## Publisher’s Note

All claims expressed in this article are solely those of the authors and do not necessarily represent those of their affiliated organizations, or those of the publisher, the editors and the reviewers. Any product that may be evaluated in this article, or claim that may be made by its manufacturer, is not guaranteed or endorsed by the publisher.
